# Nanoparticles based interventions for metal(loid) stress mitigation in plants

**DOI:** 10.1007/s44154-024-00194-6

**Published:** 2026-02-26

**Authors:** Anuj Sharma, Vaibhav Sharma, Mahipal Singh Sankhla, Kumud Kant Awasthi, Anjali Awasthi, Garima Awasthi

**Affiliations:** 1 Department of Forensic Science, PIAS, Parul University, Vadodara, Gujarat India; 2 School of Chemical & Life Sciences, Jamia Hamdard, New Delhi, India; 3 School of Basic and Applied Sciences, K.R. Mangalam University, Gurugram, Haryana India; 4 Department of Zoology, University of Rajasthan, Jaipur, Rajasthan India

**Keywords:** Metal(loid), Nanoparticle, Mitigation, Plant Stress, Toxicity

## Abstract

Metal(loid) stress is one of the key constraints limiting plant growth and productivity, thus threatening agricultural yields and ecosystem health. This review elaborates on the mechanisms through which metal(loid) stress acts on plants, with a special focus on disturbances to key physiological and biochemical aspects. Drawing on global research findings, the review then systematically discusses the interactions between various metal(loid)s and plant components, clarifying the specifity of stress responses across different plant-metal(loid) systems. A central focus of this review is the application of nanoparticles (NPs) as a mitigation strategy to enhance plant growth and improve tolerance to metal(loid) stress. Specifically, it summarizes the multifaceted roles of NPs in this context: promoting plant growth and development, inducing the activity of antioxidant enzymes, and mitigating oxidative stress. This review confirms that metal(loid) stress can strongly inhibit plant growth and physiological functions, but such adverse effects can be significantly alleviated by NPs-based interventions ultimately facilitating the cultivation of more robust and healthy plants. These findings highlight the potential of NPs-mediated strategies as a practical and effective approach to counteract metal(loid) toxicity in plants, providing valuable insights for the development of sustainable agricultural system.

## Introduction

Metal(loid) stress poses a significant challenge to agricultural system, particularly in regions with high amounts of heavy metals (HMs) such as arsenic (As), cadmium (Cd), and lead (Pb). These toxic elements not only hinder plant growth and development and reduce crop yields but also accumulate in food chains, posing severe risks to human and animal health (Khan et al. 2020). Conventional approaches for mitigating metal(loid) stress, including phytoremediation and soil amendment, have shown limited effectiveness. In recent years, however, nanoparticles (NPs) have gained increasing attention for alleviating metal(loid) stress in plants. Owing to their unique physicochemical properties, NPs can not only enhance plant growth and development by improving nutrient uptake but also reduce HM toxicity. This paper has two primary objectives: (i) to discuss the potential of NPs in reducing metal uptake by plants; (ii) to explore multiple mechanisms that NPs employ to alleviate these HM-induced stresses (Srivastava et al. 2021; Raliya et al. 2016).

The contamination of agricultural soils with metal(loid)s, such as As, Cd, Pb, and mercury (Hg), poses significant risks to plant health, crop productivity, and food safety. These toxic elements can disrupt essential physiological processes in plants, leading to retarded growth rates, decreased photosynthetic activities, and reduced nutrient absorption efficiency (Wang et al. 2016). Traditional remediation strategies, such as phytoremediation and chemical amendments, have shown limited efficacy in severely contaminated soils. In recent years, the use of NPs has gained considerable attention as a novel approach to mitigating metal(loid) stress in plants (Khan et al. 2018; Awasthi et al. 2020; Rizwan et al. 2017).

Soil is an essential part of our ecosystem, supporting all forms of life including both aquatic and terrestrial. For the growth and cultivation of plants and crops, soil is indispensable. Although the terms “soil” and “land” are often used interchangeably, it is important to recognize that land encompasses far more than just soil; coversely, soil on the other hand serves as a significant and fundmental unit of land (Tripathi et al. 2016). Soil health contributes significantly to regulating planetary processes, thereby safeguarding crop production. However, factors including accelerated industrialization, excessive application of chemical fertilizers during agricultural practices to meet the food demands of a rapidly growing population over the past decades, and other anthropogenic activities have collectively led to soil health degradation due to environmental pollution (Ma et al. 2010).

Small size and large specific surface area of NPs endow them with unique physicochemical properties, which can be exploited to enhance plant resistance against metal(loid) stress. For instance, zinc oxide nanoparticles (ZnO-NPs) and titanium dioxide nanoparticles (TiO₂-NPs) have been shown to alleviate Cd and As toxicity by promoting antioxidant enzyme activity and reducing metal uptake in plants (Rico et al. 2013). Similarly, iron oxide nanoparticles (Fe₃O₄-NPs) can immobilize Pb in the soil, thereby reducing its bioavailability and subsequent toxicity to plants (Faisal et al. 2013; Haydar et al. 2023; Sharma et al. 2016). In a study by Kumar et al. (2019), the characteristics of nanomaterials—including size, structure, and composition—play pivotal roles in their effects on plants and the environment. Specifically, nanomaterials such as graphene, graphene oxide integrated with gold nanoparticles (AuNPs), and carbon or carbon nitride nanotubes exhibited distinct influences, suggesting that these properties must be carefully considered when assessing the environmental and biological impacts of nanomaterials (Kumar et al. 2019). Additionally, Awasthi et al. (2017) highlighted the increasing prominence of green synthesis methods for NP production, primarily owing to their eco-friendly and cost-effective nature.

Several studies have also indicated that NPs improve plant growth under heavy metal (loid) stress. For instance, increased biomass production has been reported in plants exposed to Hg stress as a result of enhanced photosynthetic efficiency and nutrient uptake induced by silica NPs (Si-NPs) (Yang et al. 2017). Additionally, gene expression and stress-responsive pathways are regulated by carbon-based NPs, such as graphene oxide, consequently enhancing plant tolerance to multiple metal(loid)s (Rajput et al. 2018). Notably, NPs can serve as an alternative to conventional remediation methods for mitigating the toxic effects of metal(loid)s while ensuring sustainability in their application. Nevertheless, detailed information on the protective mechanisms of NPs remains elusive. It is essential to understand these mechanisms for optimizing the nanoparticle formulation. This paper aims to summarize the effects of different HM stresses on plants and then discuss the roles of arious NPs in mitigating such stresses, supported by the metanalysis.

## Methodology

To search the data in depth, Scopus and Google Scholar databases were consulted with a date range of 2008–2024. To retrieve documents relevant to our topic, Boolean operators were used to filter out irrelevant documents, using the following search string: ("nanoparticle" OR "nanotechnology" OR "Nano-formulation") AND ("metal stress" OR "heavy metal stress" OR "metal toxicity" OR "metal-induced stress" OR "metalloid stress" OR "metalloid toxicity" OR "metalloid-induced stress") AND ("plant" OR "crop" OR "agriculture") AND ("reduction" OR "mitigation" OR "alleviation" OR "amelioration"). Furthermore, inclusion and exclusion criteria were applied to select only relevant research articles for this review. To maintain the validity and rigor of this meta-analysis, we established clear guidelines for inclusion and exclusion. The studies included herein focus on using NPs to reduce metal(loid) stress in plants. Specifically, we considered research exploring various types of NPs, including metal oxide NPs, carbon-based nanomaterials as well as polymeric nanomaterials, with respect to their efficiency in reduction of metal (loid) toxicity. To assess stress mitigation, these reviewed studies used metrics including biomass production, root length, shoot length and chlorophyll content, among others. These studies comprised laboratory-based and field-based experiments with control and treatment groups, providing quantitative data suitable for meta-analysis. We limited our inclusion to English-language, peer-reviewed journal articles, conference papers, and theses with no restrictions on pubication date to ensure a wide range of relevant research sources. On the other hand, we excluded studies where NPs were not the primary intervention for mitigating metal(loid) stress. We also excluded research investigating stressors other than metal(loid)s, such as drought, salt, or pathogen stress. Additionally, studies lacking sufficient quantitative data, such as those without clear outcomes related to nanoparticle efficiency or plant growth, were excluded.

## Mechanisms of Metal(loid) stress in plants

Metal(loid) stress in plants occurs when plants grow in environments with high concentrations of harmful metal or metalloid ions, such as As, Cd, Pb and Hg, which can interfere with normal functioning of many physiological and biochemical processes (Faisal et al. 2013; Haydar et al. 2023; Sharma et al. 2016). To mitigate such metal stress, plants have evolved complex processes, including the activation of antioxidant systems, synthesis of phytochelatins, and vacuolar sequestration of metals. It is important to comprehend the workings of these mechanisms so that the resilience of the plant can be boosted and consequently enhance the effectiveness of strategies that involve phytoremediation (Yang et al. 2017).

### Physiological changes in Plants due to Metal(loid) stress

Heavy metals (HMs) and metalloids, including As, Cd, Pb, chromium (Cr), aluminium (Al), antimony (Sb), Hg, and nickel (Ni), exert detrimental effects on plant physiology when present at concentrations above threshold levels. These elements interfere with essential metabolic functions, leading to toxic consequences for plant growth (Yang et al. 2017). For instance, Al toxicity is a significant issue in acidic soils, where Al^3^⁺, the most toxic form of this metal, dominates under low pH conditions, affecting nearly 40% of arable land worldwide (Rajput et al. 2018; Rahman and Singh 2019). Toxic metals in plants disrupt growth by replacing essential ions that serve as cofactors or ligands for enzymes involved in primary and secondary metabolic reactions (Yang et al. 2017).

One of the primary impacts of metal(loid) stress acts on plant roots, the initial site of contact. The root apical meristem (RAM), root cap, and root tips are particularly susceptible to growth inhibition, which may result in alterations in root architectural morphology (Rajput et al. 2018). Metal ions including Cu, Pb, and Zn can displace important ions such as Ca^2^⁺ and Mg^2^⁺ from cell walls, altering structural integrity of plant tissues (Fryzova et al. 2018). Furthermore, metal(loid) exposure disrupts the structural organization of root cortex tissues, leading to thickening of cell walls, modifications in intercellular space configuration, and alterations in the development of root vasculature systems, which can further impede the efficiency of nutrient uptake (Rajput et al. 2018; Uexküll and Mutert 1995).

Stress effects extend to above-ground plant parts, including stems and leaves. Metal(loids) can impair cell division and turgor pressure in cortical and sclerenchyma cells near the phloem, thereby exerting a negative impact on overall plant growth (Uexküll and Mutert 1995; Clemens and Ma 2016). In leaves, exposure to metals such as Cd, As, and Mn can reduce leaf thickness, alter stomatal structure, and impair the structural integrity of epidermal and mesophyll cells, which in turn decrease the plant’s capacity to sustain photosynthetic efficiency (Rahman and Singh 2019; Yadav et al. 2021; Armendariz et al. 2016; Pereira et al. 2017).

### Biochemical changes in Plants due to Metal(loid) stress

At the biochemical level, heavy metal stress triggers significant oxidative stress in plants by increasing the production of reactive oxygen species (ROS). ROS, in turn, disrupts various cellular functions, particularly via interactions with sulfhydryl (-SH)-containing proteins, leading to oxidative damage. Such damage interferes with plant enzymatic functions and other metabolic activities, further contributing to the inhibition of plant growth (Fryzova et al. 2018).

Plants respond to increased ROS by activating antioxidant defence mechanisms, either through constitutive pathways or by inducing new protective responses. These adaptive strategies vary with plant species and the specific metal(loid) imposing stress (Rajput et al. 2018). However, excessive heavy methal concentrations often trigger ROS overproduction that overwhelms the plant’s antioxidant capacity, causing cellular damage (Fryzova et al. 2018).

Heavy metals also impair root meristem function, inducing physiological perturbations that inhibit root elongation and cell differentiation (Rajput et al. 2018). This disruption extends to lateral root primordia, hindering proper development and quiescent center (QC) formation (Uexküll and Mutert 1995). Similarly, root vascular tissues undergo significant changes, including the formation of dark deposits, e.g., As (III) in *Glycine max* L. (Uexküll and Mutert 1995; Clemens and Ma 2016). Moreover, the plant’s attempts to restrict the translocation of harmful metals to photosynthetic tissues can lead to changes in foliar structure, affecting photosynthetic efficiency and reducing overall plant vigor (Fryzova et al. 2018; Sharma et al. 2022).

While plants have evolved adaptive mechanisms to mitigate metal stress, e.g., adjusting the composition of root and stem tissues, metal(loid) stress remains a severe constraint on plant development, disrupting both physiological and biochemical pathways (Table 1).

**Table 1:** Toxic effect of HMs and metalloids on different plant system

HM	Inhibitory Effects on Morphology and Physiology	Crops/Plant Model	Entrance	Chelation/Translocation	Reference
Lead (Pb)	Root elongation inhibition, chlorosis, growth reduction	Wheat, Maize,	Root uptake, Foliar, Soil ingestion	Limited translocation to shoots; Complexation with organic acids	Kohli et al. 2020; Kaur et al. 2015
Cadmium (Cd)	Leaf chlorosis, reduced photosynthesis	Rice, Sunflower,	Root absorption, Foliar uptake	High mobility in xylem; Stored in vacuoles	Ma et al. 2020; Mohammadi et al. 2020
Mercury (Hg)	Root necrosis, reduced nutrient uptake	Tomato, Spinach,	Root uptake, Foliar penetration	Translocation to leaves; Low translocation to fruits	Noori et al. 2020; Velmurugan et al. 2024
Arsenic (As)	Leaf wilting, root growth inhibition	Rice, *Arabidopsis thaliana*	Soil absorption, Root uptake	Phytochelatin synthesis; Stored in root tissues	Mendoza-Cózatl et al. 2011; Briat 2010
Nickel (Ni)	Chlorosis, necrosis, inhibited root growth	Tomato, Petunia	Foliar uptake, Root absorption	Complexes with amino acids; Limited translocation to shoots	Kumar et al. 2015; Kalaivanan and Ganeshamurthy 2016
Zinc (Zn)	Reduced growth, chlorosis, inhibited enzyme activity	Mandarin (*Citrus reticulata*)*,* Peach (*Prunus persica*)	Root uptake, Foliar penetration	Chelated by organic ligands; High mobility in xylem	Song et al. 2015; Subba et al. 2014
Copper (Cu)	Leaf curling, reduced root length	Cherry Plum (*P. cerasifera*)*, *Tomato (*S. lycopersicum*)	Root absorption, Foliar application	Bound to cell walls; Translocation limited to roots	Lombardi and Sebastiani 2005; Shahbaz et al. 2010
Chromium (Cr)	Root growth inhibition, oxidative stress	Fenugreek (*Trigonella foenum graecum L.*)*,* Cauliflower (*Brassica oleracea*)	Root absorption, Soil ingestion	Low translocation; Complexation with organic acids	Chatterjee and Chatterjee 2000; Ramana et al. 2015
Aluminum (Al)	Root elongation inhibition, oxidative damage	*Arabidopsis thaliana*	Root uptake, Foliar penetration	Sequestered in root cells; Limited translocation	Kochian et al. 2015; Huang et al. 2010
Manganese (Mn)	Leaf necrosis, stunted root growth	Cucumber (*Cucumis sativus*)*,* Peach (*P. persica*)	Root uptake, Foliar penetration	Complexes with organic acids; Limited translocation to leaves	Dragišić Maksimović et al. 2012; Xue et al. 2018

## Role of nanoparticle in HM mitigation and effects on plant growth

The field of nanobiotechnology is still in its infancy stage but developing rapidly, and it has tremendous potential to augment the detoxification of metal(loid)s in the environment. NPs are increasingly being used for the remediation of metals-polluted soils, due to their efficiency, affordability, and promising application prospects, as summarized in various reported studies in Table 2. Most interactions between NPs and toxic metal(loid)s occur at the interfaces where the large suface area of NPs enables quick and efficient reactions. Their nanoscale size also makes NPs good candidates for reducing plant uptake of toxic metal(loid)s (Dragišić Maksimović et al. 2012). The primary mechanisms underlying NPs-mediated soil remediation include adsorption, immobilization or stabilization. The small size of NPss confers larger surface areas, thereby providing more active sites and lower intraparticle diffusion rates, which in turn optimize the absoption kinetics of HMs and metalloids sequestration from soil matrices (Xue et al. 2018; Martínez-Fernández et al. 2017). Consequently, NPs decrease the bioavailability of metal(loid)s to plants leading to reduced phytotoxicity associated with decreased bioaccumulation levels in plant tissues.

**Table 2:** Overview of the Efficacy of Various Nanoparticles in Mitigating HM Stress in Different Plant Models, Including Nanoparticle Size and Impact on Plant Growth

**Heavy Metal**	**Plant Species**	**Nanoparticle Type**	**Nanoparticle Size**	**Efficacy of NPs in HM Stress Mitigation**	**Effect of NPs on Plant Growth**	**References**
As	Soybean (*Glycine max* L.)	ZnO NPs combined with melatonin	46 nm	ZnO NPs reduced arsenic-induced toxicity by 26.8%, while melatonin contributed to a 17% reduction in As stress	Combined treatment completely negotiated arsenic-induced growth inhibition	Bhat et al. 2022
As	*Vigna radiata*	Iron (III) oxide nanoparticles (Fe₂O₃ NPs)	10–50 nm	Fe₂O₃ NPs (100 mg L⁻¹) significantly reduced AsO₄³⁻ toxicity, restoring normal physiological conditions	Seedling growth improved by approximately 20% compared to As-stressed plants	Shabnam et al. 2019
As	Wheat (*Triticum aestivum* L.)	Silicon oxide and iron oxide nanoparticles	–	As accumulation decreased by 54% with SiO-NPs and 40% with FeO-NPs	Improved plant vigor and biomass under arsenic stress	Manzoor et al. 2023
As	Rice (*Oryza sativa* L.)	Titanium dioxide nanoparticles (TiO₂ NPs)	–	As accumulation reduced by 40–90%, depending on nanoparticle concentration	No adverse effects on plant growth were observed	Wu et al. 2021
As	Rice (*Oryza sativa* L.)	Magnesium oxide nanoparticles with *Enterobacter* sp. strain RTN2	38–57 nm	Reduction in As stress ranged from 5.8% to 65.5% at 50, 100, and 200 mg kg⁻¹ NPs in soil	Plant height increased by 18.7%, 25.2%, and 32.7% at respective concentrations	Ahmed et al. 2021
Cr	Rice (*Oryza sativa*)	Silicon nanoparticles (Si NPs)	–	Cr accumulation decreased by 32.7% following Si NPs application	Shoot length increased by 35.2%, and root length by 21.2%	Sharma et al. 2022
Cr	*Pisum sativum* (L.)	Silicon nanoparticles	75–125 nm	Cr accumulation reduced to 516.6 ± 14.1 mg kg⁻¹ in roots and 35.2 ± 1.5 mg kg⁻¹ in shoots	Significant improvement in overall growth parameters of pea seedlings	Tripathi et al. 2015
Cd	Barley	Calcium oxide nanoparticles (CaO NPs)	10–24 nm	Cd content reduced by 29.7–39.1% in roots and 42.5–68.4% in shoots across two genotypes (LJZ and Pu-9)	Improved tolerance and healthier plant development	Nazir et al. 2022
Cd	Rapeseed (*Brassica napus* L.)	Silicon nanoparticles	14–35 nm	Root and shoot Cd uptake reduced by 25.4% and 33.3%, respectively, at 250 mg kg⁻¹ Si	Plant growth parameters remained unaffected	Ahmed et al. 2023
				NPs		
Cd	*Leucaena leucocephala* seedlings	ZnO nanoparticles	2–64 nm	Significant reduction in Cd-induced stress	Shoot and root growth increased by 27.2% and 35.0%, respectively	Venkatachalam et al. 2017
Cd	Wheat (*Triticum aestivum* L.)	Silicon nanoparticles	–	Cd concentration reduced by 16–58% in shoots, 19–64% in roots, and 20–82% in grains	Spike length increased by 54% (soil application) and 61% (foliar application)	Ali et al. 2019
Cd	*Pleioblastus pygmaeus*	24-Epibrassinolide and TiO₂ nanoparticles	–	Combined treatment alleviated Cd toxicity effectively	Overall plant growth increased by 39% compared to Cd-stressed plants	Emamverdian et al. 2022

NPs have been increasingly used in a growing body of evidence to limit arsenic (As) mobility and bioavailability in crops, thereby reducing As bioaccumulation and associated toxic effects. NPs can be deployed to manage As mobilization, translocation, as well as bioaccumulation in diverse plant species. Anthropogenic As release into the environment has become a global concern, with several studies indicating that NPs may help mitigate As phytotoxicity and its accumulation in crops (Tan et al. 2012; Dixit et al. 2015; Ahmed et al. 2021a). In particular, metal oxide NPs have received considerable attention for their unique design and fabrication process. With numerous adsorption sites and high specificity for As, these NPs interact with As ions leading to As precipitation, thus reducing As availability for plant uptake and translocation (Wu et al. 2020).

Research on the effects of ZnO-NPs on plant species remains limited (Praveen et al. 2018; Habuda-Stanić and Nujić 2015). However, ZnO-NPs are known for their distinct optical and electrical properties, which make them useful for diverse applications, such as coatings used to remove toxic compounds or biogenic substances including HMs or metalloids (Stampoulis et al. 2009). Additionally, ZnO-NPs have recently been utilized as nanofertilizers in zinc-deficient areas worldwide (Lin and Xing 2007; Behnajady et al. 2006; Milani et al. 2012). Various studies have revealed that ZnO-NPs have positive effects on plant growth, physiology as well as secondary metabolism. For example, in mung bean (Mahajan et al. 2011) and cotton (Venkatachalam et al. 2017), optimal concentrations of ZnO-NPs were found to enhance plant growth. Wang et al. (2018) observed that ZnO-NP treatment significantly reduced total As bioaccumulation in rice seedlings—by 72% for As(III) and 68% for As(V)—in comparison with plants treated soly with As(III) and As(V). Similarly, Singh et al. (2013) demonstrated the potential of ZnO-NPs to adsorb and remove As from water sources. More recently, Yan et al. (2021) and Kumar et al. (2021) confirmed that application of ZnO-NPs effectively reduced As toxicity in paddy and wheat seedlings by inhibiting As bioaccumulation (Kumar et al. 2021; Yan et al. 2021). Such findings indicate that adsorption of the metalloid onto NP surfaces, combined with slow release of Zn ions into plant cytoplasm, results in decreased As content in NP-treated plants. Akin to this, Zeeshan et al. (2021) further confirmed that soybean is also amenable to ZnO-NP-mediated As mitigation, where their study showed that ZnO-NPs supplementation reduced As content in both root and shoot tissues compared with control groups.

Silicon (Si), a metalloid, is recognized for its beneficial effects on plant development and health. This is attributed to Si being considered as a quasi-essential mineral element, that enhances optimum plant growth. In traditional agriculture, Si-NPs have been utilized for crop improvement, sustainable agricultural practices, and stress protection (Bhat et al. 2021; Roychoudhury 2020; Rastogi et al. 2019). While Si-NPs have gined popularity in agriculture research on their role in mitigating metal(loid) toxicity in plants remains limited (Hussain et al. 2020). Earlier work by Li et al. (2009) indicated that fertilization with Si could effectively reduce As contamination in rice straw and grains (Li et al. 2009). This decrease is linked to the competitive interaction between Si and As during uptake into the root cells and transportation from root to aerial parts. Thus, Si-NPs could serve as an effective strategy for alleviating As toxicity in crops, warranting further investigation regarding their possible agricultural applications.

**Fig. 1:**
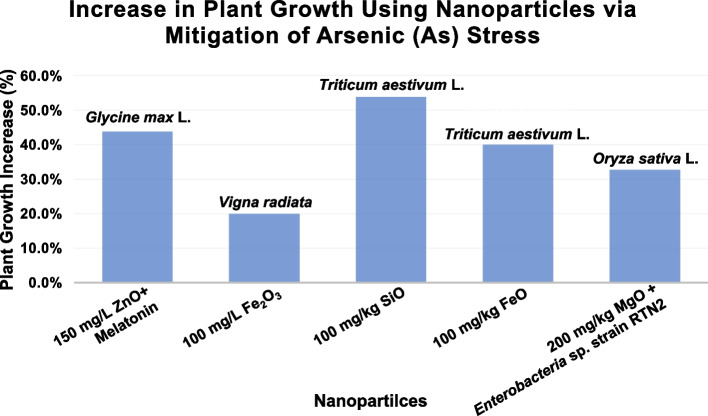
Plant growth promotion after mitigation of Arsenic stress using various nanoparticles. Datas were collected and and analyzed from previous studies (Bhat et al. 2022; Shabnam et al. 2019; Manzoor et al. 2023; Ahmed et al. 2021)

Bhat et al. (2022) investigated the combined use of ZnO-NPs and melatonin in soybean *(Glycine max *L*.)* to mitigate As stress (Bhat et al. 2022). Their foundings showed that As stress significantly inhibited plant growth (~ 34%), reduced photosynthesis-related parameters (~ 18–28%), and induced reactive oxygen species (ROS) accumulation. However, the combined application of melatonin and ZnO-NPs completely negated the adverse impacts of As, resulting in a 37% increase in plant height compared to plants treated with As alone. Shabnam et al. (2019) studied the effects of iron (III) oxide NPs (Fe_2_O_3_-NPs) on *Vigna radiata* under As stress. Their results showed that As exposure reduced shoot length and dry biomass by approximately 8% and 12%, respectively. However, the application of 100 mg/L Fe_2_O_3_-NPs reduced the As-induced decline in root length from 75% to 20%, with complete recovery observed at higher nanoparticle concentrations (200 and 400 mg/L). Manzoor et al. (2023) explored the efficacy of SiO-NPs and FeO-NPs in wheat (*Triticum aestivum *L*.*) grown in As-contaminated soils. They found that nanoparticle application significantly improved plant growth: SiO-NPs increased plant height by up to 54%, FeO-NPs by 40%, and FeO-NPs also increased dry weight by 52%. Furthermore, FeO-NPs significantly reduced As translocation in plants, with reductions of 74% in roots, 54% in shoots, and 78% in grains compared to the control. SiO-NPs showed slightly lower reduction efficiency. Wu et al. (2021) investigated the application of TiO_2_-NPs in rice seedlings (*Oryza sativa *L*.*) to reduce As bioaccumulation. Their study showed that TiO_2_-NPs could reduce As bioaccumulation by 40% to 90%, depending on treatment conditions, without adverse effects on plant growth. This reduction was attributed to the strong sorption apacity of TiO_2_-NPs. Ahmed et al. (2021) focused on the use of magnesium oxide nanoparticles (MgO-NPs), that were synthesized from a native *Enterobacter* sp. strain RTN2, to mitigate As stress in rice (*Oryza sativa* L.). They observed a concentration-dependent improvements in plant height, with increases of 18.7%, 25.2%, and 32.7% at MgO-NPs concentrations of 50, 100, and 200 mg/kg soil, respectively. Additionally, MgO-NPs reduced root-to-shoot As translocation by 5.8%, 30.7%, and 65.5% at the corresponding concentrations. Farooq et al. (2022) studied the mitigation effects of melatonin-selenium nanoparticles (MT-Se NPs) on As-induced stress in* Brassica napus*. Their results revealed a substantial reduction in As accumulation in leaves and roots. Specifically, leaf As content was reduced by 76.81% with 0.05 g MT-Se NPs and 73.73% with 0.02 g MT-Se NPs. The root As content decreased by 55.35% and 46.6% at the same respective concentrations (Farooq et al. 2022). Collectively, these studies highlight the potential of various nanopartiles to alleviate As stress in different plant species, offering promising strategies for enhancing crop resilience in As-contaminated environments. The significant recovery of plant growth after the mitigation of As using nanoparticles can be seen in Fig. 1.

**Fig. 2:**
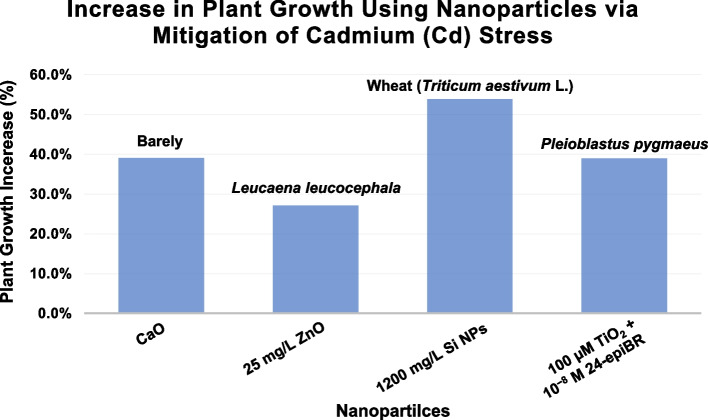
Plant growth promotion after Cadmium stress alleviation via arious NPs. Datas were collected and and analyzed from previous studies (Nazir et al. 2022; Venkatachalam et al. 2017; Ali et al. 2019; Emamverdian et al. 2022)

Nazir et al. (2022) examined the effects of calcium oxide nanoparticles (CaO-NPs) on cadmium (Cd) stress in barley (*Hordeum vulgare*). They observed a significant reduction in Cd content in both roots and shoots of two barley genotypes (LJZ and Pu-9), with decreases of 29.7% and 39.1% in roots and 42.5% and 68.4% in shoots, respectively. Despite Cd-induced growth inhibition, CaO-NPs treatment mitigated these effects: root and shoot length inhibition were reduced by 29.1% and 36.7% in genotype LJZ and 5.6% and 32.8% in genotype Pu-9, respectively (Nazir et al. 2022). Ahmed et al. (2023) investigated the impact of silicon nanoparticles (Si-NPs) on rapeseed (*Brassica napus *L*.*) under Cd stress. They reported that the application of 250 mg/kg Si-NPs led to a maximum reduction in Cd uptake: 25.4% in roots and 33.3% in shoots, compared to the Cd-treated group. Additionally, Si-NPs significantly enhanced the fresh and dry weights of rapeseed plants by 33.3% and 32.6%, respectively, demonstrating their effectiveness in promoting plant growth under Cd stress (Ahmed et al. 2023). Venkatachalam et al. (2017) focused on *Leucaena leucocephala* seedlings exposed to Cd stress. Their study found that the addition of ZnO NPs in the presence of HMs, including Cd, resulted in a substantial increase in shoot and root growth. Specifically, at a Cd concentration of 50 mg/L, 25 mg/L ZnONPs increased shoot and root growth by 27.2% and 35%, respectively (Venkatachalam et al. 2017). Ali et al. (2019) explored the application of Si NPs through foliar spray in wheat (*Triticum aestivum* L.) to combat Cd stress. The study revealed that Si NPs treatment reduced Cd content by 16-58% in shoots, 19-64% in roots, and 20-82% in grains, respectively. Additionally, the spike length was significantly enhanced by 54% in soil-applied Si NP treatments and 61% in foliar treatments, compared to unamended controls, indicating improved plant growth under Cd stress (Ali et al. 2019). Emamverdian et al. (2022) examined the combined effects of 24-epibrassinolide and titanium oxide (TiO_2_) NPs on *Pleioblastus pygmaeus* under Cd stress. Their findings showed that plant growth was increased by 39%, demonstrating the potential of these NPs to alleviate Cd-induced growth inhibition (Emamverdian et al. 2022). Koleva et al. (2022) studied the combined use of iron oxide and silicon nanoparticles (IONPs+SiNPs) in *Phaseolus vulgaris* exposed to Cd stress. The results indicated that combined treatment with FeO NPs and Si NPs significantly increased shoot fresh weight by 45% and root fresh weight by 19%, highlighting their synergistic effect in enhancing plant growth under Cd stress (Koleva et al. 2022). Overall, these studies underscore the efficacy of various NPs in mitigating Cd stress in different plant species, facilitating better growth and reduced Cd accumulation, thereby offering a viable approach for enhancing plant resilience in Cd-contaminated environments (Fig. 2).

Sharma et al. (2022) investigated the effect of Si NPs on *Oryza sativa* (rice) exposed to Cr (VI). They reported Cr (Ⅵ) stress caused a substantial reduction in plant length, with shoot and root lengths decreased by 36.4% and 34.6%, respectively (Sharma et al. 2022). The stress also led to a significant decline in photosynthetic pigments, with total chlorophyll content reduced by 37.9%, and carotenoids by 29.3% compared to the control. However, the combination of Si NPs and indole-3-acetic acid (IAA) mitigated these adverse effects, limitinging the decline in total chlorophyll and carotenoids content to just 7.5% and 9.8%, respectively. This finding suggests that Si NPs, especially when used in conjunction with IAA, can significantly protect the photosynthetic machinery of rice under Cr (Ⅵ) stress.

In another study focusing on Cr stress mitigation, Tripathi et al. (2015) examined *Pisum sativum* (pea) exposed to Cr (VI). Cr (Ⅵ) stress led to substantial reductions in root and shoot fresh weights by 47% and 34%, respectively, and dry weights by 36% and 21%. Additionally, total chlorophyll and carotenoids content decreased by 18% and 12%, respectively, under Cr (Ⅵ) exposure. However, when Si NPs were introduced, the decline in photosynthetic efficiency parameters (F_v_/F_m_, F_v_/F_0_, and qP) was minimized to only 8%, 2%, and 3%, respectively, indicating a significant protective effect of Si NPs on photosynthetic system of pea plants under Cr (Ⅵ) stress (Tripathi et al. 2015). In a separate investigation on wheat (*Triticum aestivum*), researchers examined the role of Fe_2_O_3_ NPs on wheat (*Triticum aestivum)*) under the stress of 350 ppm Cr. Cr exposure led to significant reductions in shoot and root lengths by 18% and 24%, respectively, and in weights by 18% and 26%. Moreover, a notable decrease in total chlorophyll content was observed (Zafar et al. 2024). When Fe_2_O_3_ NPs were applied, there was an increase in chlorophyll content compared to Cr-stressed plants without NPs, however, this increase was less than that observed in plants treated only with Fe_2_O_3_ NPs at a concentration of 450 ppm. This indicates that Fe_2_O_3_ NPs can partially alleviate the adverse effects of Cr on chlorophyll content, although their efficacy may vary depending on the concentration used. In conclusion, Si NPs and FeO NPss demonstrate substantial potential in mitigating the harmful effects of Cr on plant growth and photosynthetic efficiency. Si NPs, particularly when combined with IAA, appear highly effective in preserving chlorophyll and carotenoid content under Cr stress, while Fe_2_O_3_ NPs offer a moderate protective effect. These findings underscore the value of nanoparticle applications in enhancing plant resilience in Cr-contaminated environments.

## Nanoparticles' influence on chlorophyll content

In photosynthetic antenna systems, chlorophylls are vital molecules that facilitate the absorption of solar energy, as well as charge separation and electron transport in reaction centers. The highly coordinated process of chlorophyll metabolism relies on multiple cooperative biochemical pathoways supported by many enzymes (Tanaka and Tanaka 2006; Beale 1999). Generally, chlorophyll metabolism is defined as a process that synthesizes or degrades chlorophyll molecules to meet cellular demands (Beale 1999).

In non-vascular plants like bryophytes and lichens, HMs at hazardous concentrations have been shown to cause membrane damage, ion leakage, and reduced chlorophyll concentrations (Welschmeyer 1994; Brown and Wells 1990; Garty et al. 1992). In this regard, Shakya et al. (2008) reported that at the treatment concentration of 10^–6^ M, the Cu content was 15 mg/kg in *Thuidium delicatulum*, 18 mg/kg in *Thuidium sparsifolium* and 47 mg/kg in *Ptychanthus striatus*. When exposed to higher levels (10^–4^ M), significant decreases of total chlorophyll were observed: 54% in *T. delicatulum*, 22% in *T. sparsifolium,* and 42% in *P. striatus*. Total chlorophyll (Chl-s) decreased negligibly with increasing Zn or Pb concentration on two species; however, total chlorophyll content in nearly all primary leaves decreased significantly after treatment with copper chloride (CuCl_2_). At CuCl_2_ concentrations of 0.1 mM, 0.2 mM, and 0.3 mM, total chlorophyll content dropped by only 14%, 16%, or less. Additionally a loss between 5% to 12% in total Chl-a upon exposure to lead nitrate was also recorded (Shakya et al. 2008). The loss occurred when Pb(NO_3_)_2_ was applied at concentrations ranging from 4 to 20 mM. When primary leaves were treated with lead acetate (Pb(CH_3_COO)_2_) at doses of 0.1 to 0.4 mM, total chlorophyll content decreased by 1-12%. For bean (*Phaseolus vulgaris* L.) seedlings, total chlorophyll content in primary leaves decreased with increasing Cd concentration, showing a 18.3–22.5% reduction at 0.05, 0.06, and 0.08 mM Cd. Under treatments with 0.02, 0.04, and 0.06 mM HgCl_2_, total chlorophyll content decreased by 24.9–29.2%.

The mobility and bioavailability of HMs, including their absorption and transformation in plants, can be reduced by NPs (Zengin and Munzuroglu 2005). Similarly, Zafar et al. has found that biosynthesized Fe_2_O_3_-NPs could alleviate Cr stress in wheat (*Triticum aestivum*) (Zafar et al. 2024). These findings indicated that Cr has negative effects on the plants by reducing overall chlorophyll levels, while Fe_2_O_3_-NPs mitigate the detrimental impacts of HMs by enhancing photosynthetic capacity. For soybean plants, the SPAD index, an indicator of chlorophyll content, was significantly affected by As toxicity. As-treated plants showed a 28.6% decrease in SPAD values compared to controls. However, when melatonin or ZnO-NPs were applied alongside As treatment, the SPAD index increased by 21.10% and 29.40%, respectively (Bhat et al. 2022). For rice, chlorophyll content in leaves exposed to As at 2 mg/L was significantly lower than that in controls (*p* < 0.05) with a reduction rate of 27.3%. Following treatment with ZnO NPs, Yan et al. reported a significant increase in chlorophyll concentration by 2.7–90.3% (Yan et al. 2021).

## Reduction of oxidative stress using nanoparticles

Superoxide radical (ROS), hydrogen peroxide (H_2_O_2_), hydroxyl radical (•OH), and singlet oxygen (^1^O_2_) are among the naturally occurring byproducts of metabolic processes. They are produced in a variety of cell organelles, including the plasma membrane, mitochondria, peroxisomes, and chloroplasts (Zhou et al. 2020). The production of ROS and the ensuing dysregulation of cellular redox homeostasis are closely associated with the toxic effects of HMs. Under prolonged environmental stress, plants generate excessive ROS, which cannot be completely scavenged by the active oxygen scavenging system (Noor et al. 2022; Meng et al. 2018). Consequently, ROS interacts with lipids to produce lipid peroxidation, denatures the proteins essential for cellular functioning, and ultimately impairs DNA and cellular integrity. Oxidative stress in plants arises either from the direct effects of environmental stress or the indirect effects of excessive ROS production—which disrupts plant structural integrity. To cope with such stress, plants have evolved a variety of adaptive mechanisms. Notably, the levels of oxidative damage and lipid peroxidation in plants may be assessed by measuring the contents of malondialdehyde (MDA) (Rajput et al. 2018; Paithankar et al. 2021; Flora et al. 2008).

Enhancing HM tolerance in plants can be achieved by reducing HM accumulation and improving photosynthetic efficiency. Sun et al. (2023) investigated the effects of Cd stress on tomato sprouts and reported that Cd stress increased MDA levels by 104.4% in leaves and 116.8% in the roots compared to the control (Sun et al. 2023). Amir et al. (2024) examined the dose-dependent effects of sulfur-doped gold NPs (S-Au NPs) on plant growth and morphology. The study indicated that higher concentrations (250 and 300 mM) of S-AuNPs were detrimental, leading to oxidative stress via overproduction of ROS. This stress further inhibitted plant growth, reduced photosynthetic efficiency, and impaired key morphological traits. However, an optimal concentration of 150 mM S-AuNPs significantly enhanced plant growth parameters, demonstrating a beneficial threshold for the application of these NPs in plant systems (Amir et al. 2024). Ahmad et al. demonstrated that foliar spraying of ZnO NPs under Cd stress reduced MDA levels in tomato seedling leaves and roots by about 34% and 29.4%, respectively, compared to the untreated control. Additionally, this treatment significantly enhanced catalase (CAT), an enzyme that facilitates the decomposition of H_2_O_2_ into O_2_ and H_2_O, in tomato seedlings: CAT activity increased by 57.8% in leaves and 116.6% in roots, which was higher than that observed from the single Cd treatment group (Ahmad et al. 2020). However, Ahmad et al. (2020) also noted that for plants treated with 10 µM As, the exogenous application of ZnO NPs at doses of 50 mg/L or 100 mg/L resulted in an increase in H_2_O_2_ content by approximately 70.69% or 30.69%, respectively, compared to the As-untreated control plants (as explained above). By contrast, this effect was not observed in other treatment groups: for example, in As-untreated plants, there was no significant difference in H_2_O_2_ content among groups, as all these groups exhibited similar H_2_O_2_ levels. Ahmad et al. (2020) further pointed out that such results may vary depending on the applied As concentration. Additionally, they mentioned that a one-step process—involving the synthesis of ZnO NPs followed by decoration with surfactants—could yield spherical nanoparticles with controlled sizes and narrow size distributions, which is consistent with findings reported in other studies (Dixit et al. 2015).

## Conclusion

The nanotechnology revolution has opened up novel strategies for tackling metal(loid) stress, a widespread issue that constrains agricultural productivity and food security. Engineered NPs represent an innovative, multidimensional approach that confers significant benefits by alleviating the phytotoxic impact of toxic elements including As, Cd and Pb. Notably, metal oxide NPs such as ZnO, TiO₂ and Fe₃O₄—among others—effectively decrease bioavailability of hazardous metal(loid)s in soil–plant system, mitigating their adverse effects on plants at molecular level. In this regard, recent studies have revealed that interactions between nanoparticulate materials and plant roots contribute to decreased phytotoxicity primarily by restrcting the transportation of metal(loid)s from roots to shoots. Moreover, these nanoscale structures can manipulate the expression of specific stress-responsive genes, including those governing ROS detoxification pathways where antioxidant enzymes such as SOD or CAT get activated during metal(loid) stress responses. Moreover, the integration of NPs into plant systems has been shown to enhance nutrient uptake and utilization efficiency, consequently augmenting plant resilience to environmental stressors. Additionally, the synergistic application of NPs with other agronomic practices, such as organic amendments and microbial inoculants, has emerged as a focal point of research, offering potential pathways for developing integrated strategies to bolster crop protection and improved yield. However, the field of agricultural nanotechnology remains in its infancy stage, necessitating sustained research efforts to clarify the long-term ecological effects and safety implications associated with NPs usage in agriculture. Specifically, the interactions between NPs and soil microbiota, as well as NPs environmental fate (e.g., their accumulation, persistence) require comprehensive characterization for sustainable nanotechnology deployment. Furthermore, the development of cost-effective, large-scale NP synthesis protocols is imperative for facilitating the translation of nanotechnology into mainstream agricultural practices.

## Data Availability

Though all data is presented in the tables. The datasets generated and/or analysed during the current study will be made available on request.

## Abbreviations

NPs: Nanoparticles
Fe₃O₄: Fe₃O₄ Iron Oxide
ZnO: Zinc Oxide
TiO₂: Titanium dioxide
As: Arsenic
Cd: Cadmium
Pb: Lead
Cr: Chromium
Al: Aluminum
Sb: Antimony
Hg: Mercury
Ni: Nickel
